# Supplementary material for: Insight into the Wnt Pathway in Sporadic Small Bowel Adenocarcinoma
Source: Cancers (Basel). 2025 Sep 10;17(18):2965. doi: 10.3390/cancers17182965 (PMC12468334; doi:10.3390/cancers17182965)
Supplement: Supplementary file 1 [file cancers-17-02965-s001.zip › Supplementary Material.docx]

**SUPPLEMENTARY MATERIAL**

| Element | Title | Page |
| --- | --- | --- |
| Table S1 | Antibodies used for immunostaining | 2 |
| Table S2 | Immunoreactivities of the mucin proteins, cyclin D1, c-Myc, β-catenin, E-cadherin and Wnt5a scored in accordance with the percentage of unequivocally positive epithelial cells | 3 |
| Table S3 | Raw next-generation sequencing data | 4 |
| Table S4 | Relationship between common genetic alterations and clinicopathological factors of patients with SBA (N=48) | 8 |

Table S1. Antibodies used for immunostaining

| Antibody | Clone | Dilution rates | Buffer (pH) for microwave | Source |
| --- | --- | --- | --- | --- |
| MLH-1 | G168-15 | 1:10 | High | Biosciences |
| MSH-2 |  | 1:50 | High | Dako |
| MSH-6 |  | 1:100 | Low | Leica |
| PMS-2 | EP51 | RTU | High | Leica |
| β-catenin |  | 1:250 | Low | Abcam |
| Cyclin D1 | SP4 | 1:100 | Low | Thermo Fisher |
| C-Myc | Y69 | 1:100 | High | Abcam |
| E-cadherin | NCH-38 | 1:50 | Low | Dako |
| CD10 | 56C6 | 1:100 | Low | Leica |
| MUC2 | CCP58 | 1:50 | High | Dako |
| MUC5AC | CLH2 | 1:50 | Low | Dako |
| MUC6 | CLH5 | RTU | Low | Dako |
| Wnt5a |  | 1:400 | Low | Abcam |

Table S2. Immunoreactivities of the mucin proteins, cyclin D1, c-Myc, β-catenin, E-cadherin and Wnt5a scored in accordance with the percentage of unequivocally positive epithelial cells

|  | Cyclin D1 | C-Myc | β-catenin | E-cadherin | Wnt5a |
| --- | --- | --- | --- | --- | --- |
| 0 (<10%) | 30 (40) | 44 (59) | 19 (25) | 10 (13) | 41 (55) |
| 1+ (10-25%) | 21 (28) | 18 (24) | 0 | 7 (9) | 19 (25) |
| 2+ (26-50%) | 13 (17) | 7 (9) | 7 (9) | 12 (16) | 8 (11) |
| 3+ (51-75%) | 5 (7) | 0 | 1 (1) | 4 (5) | 3 (4) |
| 4+ (75% <) | 6 (8) | 6 (8) | 48 (64) | 42 (56) | 4 (5) |
| Data are given as number (%) of positive cases | | | |  |  |

Table S3. Raw next-generation sequencing data

| Specimen | | Gene ID | | Mutation ID | Frequency | AA Mutation | CDS Mutation |
| --- | --- | --- | --- | --- | --- | --- | --- |
| 1902478 | | KRAS | | 554 | 41.2 | p.Q61H | c183A>C |
|  | GNAS | | | 27887 | 21.2 | p.R844C | c.2530C>T |
| 1900971 | GNAS | | | 27887 | 14.4 | p.R844C | c.2530C>T |
| 2109194 | GNAS | | | 27896 | 77.73 | Q870R | 2609A>G |
| 2308104 | No hotspot | | |  |  |  |  |
| 2311086 | SMAD4 | | | 4382822 | 13.16 | G508D | 1523G>A |
| 400406 | TP53 | | | 22908 | 4.4 | p.unknown | c.376-1G>T |
| 2007118 | No hotspot | | |  |  |  |  |
| 1911740 | ERBB4 | | | 9583227 | 43.64 | unknown | 421 + 58A>G |
|  | KDR | | | 149773 | 56.09 | Q472H | 1416A>T |
|  | TP53 | | | 2500061 | 93.65 | P72R | 215C>G |
| 1905473 | ERBB4 | | | 9583227 | 45.30 | unknown | 421 + 58A>G |
|  | PIK3CA | | | 6834758 | 21.50 | unknown | 352 + 40A>G |
|  | KDR | | | 8870412 | 38.85 | unknown | 798 + 54G>A |
|  | APC | | | 18852 | 10.35 | R876 | 2626C>T |
|  | EGFR | | | 1451600 | 39.97 | Q787 | 2361G>A |
|  | RET | | | 4418405 | 79.18 | L769 | 2307G>T |
|  | HRAS | | | 249860 | 40.60 | H27 | 81T>C |
| 2005337 | KRAS | | | 532 | 36.3 | p.G13D | c.38G>A |
|  | GNAS | | | 27887 | 31.4 | p.R844C | c.2530C>T |
| 2010966 | No hotspot | | |  |  |  |  |
| 2107879 | No hotspot | | |  |  |  |  |
| 2308105 | No hotspot | | |  |  |  |  |
| 2005580 | No hotspot | | |  |  |  |  |
| 2012362 | KRAS | | | 521 | 24.42 | G12D | 35G>A |
|  | TP53 | | | 10735 | 37.68 | R213Q | 638G>A |
| 395363 | TP53 | | | 10735 | 10.6 | p.R213Q | c.638G>A |
|  | TP53 | | | 11059 | 4.6 | p.C238Y | c.713G>A |
|  | TP53 | | | 44033 | 11.2 | p.T155I | c.464C>T |
|  | TP53 | | | 44391 | 8.1 | p.Y163= | c.489C>T |
|  | TP53 | | | 45479 | 6.5 | p.H168= | c.504C>T |
| 1910422 | KRAS | | | 520 | 7 | p.G12V | c.35G>T |
| 2301729 | BRAF | | | 452 | 17.10 | G466A | 1397G>C |
| 1503936 | TP53 | | | 99729 | 41 | p.R273H | c.818G>A |
| 2006106 | No hotspot | | |  |  |  |  |
| 444476 | APC | | | 13125 | 16.2 | p.R1114* | c.3340C>T |
|  | TP53 | | | 44599 | 1.9 | p.R196Q | c.587G>A |
| 420756 | KIT | | | 28026 | 28.5 | p.M541L | c.1621A>C |
|  | PIK3CA | | | 163484 | 2.9 | p.E80K | c.238G>A |
|  | TP53 | | | 44603 | 3.2 | p.G279R | c.835G>A |
|  | TP53 | | | 43955 | 2 | p.V172I | c.514G>A |
| 1164845 | TP53 | | | 10771 | 2.7 | p.P250L | c.749C>T |
|  | TP53 | | | 43704 | 2.3 | p.C135= | c.405C>T |
|  | TP53 | | | 44300 | 6.2 | p.S183L | c.548C>T |
|  | TP53 | | | 44971 | 5.7 | p.H178= | c.534C>T |
|  | TP53 | | | 45103 | 2.7 | p.K164= | c.492G>A |
|  | TP53 | | | 45622 | 5.7 | p.G154D | c.461G>A |
|  | TP53 | | | 45627 | 4.8 | p.I162= | c.486C>T |
|  | TP53 | | | 45671 | 3 | p.R174M | c.521G>T |
| 463014 | BRAF | | | 467 | 11.2 | p.D594G | c.1781A>G |
|  | KIT | | | 28026 | 53.5 | p.M541L | c.1621A>C |
| 403049 | KRAS | | | 517 | 51.4 | p.G12S | c.34G>A |
|  | TP53 | | | 43871 | 7.2 | p.R249M | c.746G>T |
| 1508982 | CDKN2A | | | 12473 | 17.8 | p.R58* | c.172C>T |
|  | PIK3CA | | | 775 | 21.6 | p.H1047R | c.3140A>G |
|  | PIK3CA | | | 764 | 2.3 | p.E545G | c.1634A>G |
| 374800 | APC | | | 18852 | 20.8 | p.R876* | c.2626C>T |
|  | APC | | | 18734 | 12.8 | p.T1556Nfs*3 | c.4666dup |
|  | TP53 | | | 44225 | 3.5 | p.E287K | c.859G>A |
|  | TP53 | | | 10705 | 2.7 | p.R196* | c.586C>T |
| 384578 | APC | | | 41616 | 23.8 | p.E1552* | c.4654G>T |
|  | PIK3CA | | | 774 | 24.1 | p.H1047Y | c.3139C>T |
|  | TP53 | | | 11183 | 3.5 | p.R267W | c.799C>T |
| 412854 | FBXW7 | | | 22932 | 7.8 | p.R465C | c.1393C>T |
|  | APC | | | 18734 | 7.2 | p.T1556Nfs*3 | c.4666dup |
|  | KIT | | | 17946 | 4.6 | p.E562K | c.1684G>A |
|  | TP53 | | | 11148 | 3.3 | p.A159V | c.476C>T |
|  | TP53 | | | 44036 | 1.5 | p.S99F | c.296C>T |
| 399402 | CTNNB1 | | | 5667 | 2.5 | p.S45F | c.134C>T |
|  | TP53 | | | 99933 | 3.2 | p.R273C | c.817C>T |
|  | TP53 | | | 129851 | 2 | p.H179Y | c.535C>T |
|  | TP53 | | | 129859 | 4 | p.P152L | c.455C>T |
| 375483 | TP53 | | | 43903 | 53.3 | p.V157G | c.470T>G |
|  | KRAS | | | 554 | 51.4 | p.Q61H | c.183A>C |
| 1702432 | CTNNB1 | | | 1717883 |  | p.A21_A152del | c.60_455del396 |
|  | BRAF | | | 272639 | 24.6 | p.D594N | c.1780G>A |
| 1707847 | KRAS | | | 520 | 23.9 | p.G12V | c.35G>T |
|  | TP53 | | | 10808 | 33.4 | p.Y163C | c.488A>G |
| 376896 | TP53 | | | 11059 | 32.6 | p.C238Y | c.713G>A |
|  | TP53 | | | 43700 | 41.3 | p.C238S | c.712T>A |
|  | TP53 | | | 45677 | 35.8 | p.C238* | c.714T>A |
|  | GNAS | | | 123397 | 2.7 | p.R844C | c.2530C>T |
| 383202 | APC | | | 19721 | 20.79 | Ser1465ArgfsTer9 | 4393_4394dup (Insertion) |
|  | EGFR | | | 1451600 | 60.89 | Q787= | 2361G>A |
|  | HRAS | | | 249860 | 98.10 | H27 | 81T>C |
|  | TP53 | | | 10735 | 35.93 | R213Q | 638G>A |
|  | TP53 | | | 2500061 | 96.80 | P72R | 215C>G |
|  | SMAD4 | | | 14122 | 34.72 | R361H | 1082G>A |
|  | | | SMARCB1 | 9056833 | 26.48 | I1204 | 612C>T |
| 1710891 | | | KRAS | 521 | 15.9 | p.G12D | c.35G>A |
|  | | | TP53 | 6932 | 29.3 | p.G245S | c.733G>A |
|  | | | TP53 | 43776 | 3.3 | p.E287= | c.861G>A |
| 391146 | | | CTNNB1 | 22566 | 2.2 | p.V22I | c.64G>A |
|  | | | KIT | 33966 | 2.9 | p.L576F | c.1726C>T |
|  | | | KRAS | 554 | 36 | p.Q61H | c.183A>C |
|  | | | TP53 | 43903 | 51.6 | p.V157G | c.470T>G |
| 390766 | | | TP53 | 10886 | 23.6 | p.Q104* | c.310C>T |
|  | | | TP53 | 43657 | 22.2 | p.P190L | c.569C>T |
|  | | | TP53 | 99721 | 22 | p.R342* | c.1024C>T |
|  | | | TP53 | 10771 | 5.1 | p.P250L | c.749C>T |
|  | | | TP53 | 46074 | 4.1 | p.R202C | c.604C>T |
|  | | | KIT | 19110 | 13.3 | p.V825I | c.2473G>A |
|  | | | CDKN2A | 12501 | 12.7 | p.G122D | c.365G>A |
|  | | | CDKN2A | 12484 | 12.5 | p.D108N | c.322G>A |
|  | | | CDKN2A | 13496 | 9.2 | p.V51I | c.151G>A |
|  | | | STK11 | 21359 | 11.1 | p.E199K | c.595G>A |
|  | | | PIK3CA | 760 | 7.7 | p.E542K | c.1624G>A |
|  | | | PTEN | 5153 | 7.7 | p.Q17* | c.49C>T |
|  | | | VHL | 14408 | 7.3 | p.C162Y | c.485G>A |
|  | | | NOTCH1 | 12776 | 2.9 | p.Q2459* | c.7375C>T |
|  | | | EGFR | 6240 | 2.4 | p.T790M | c.2369C>T |
| 397245 | | | No hotspot |  |  |  |  |
| 2311481 | | | ATM | 21624 | 26.80 | R3047 | 9139C>T |
|  | | | KRAS | 517 | 27.61 | G12S | 34G>A |
|  | | | SMAD4 | 373800 | 53.70 | D351G | 1052A>G |
| 2312260 | | | PIK3CA | 776 | 27.20 | H1047L | 3140A>T |
|  | | | PTEN | 5317 | 31.87 | Y65 | 195C>G |
|  | | | KRAS | 87301 | 10.46 | A11_G12dup | 30_35dup(insertion) |
| 1904298 | | | BRAF | 451 | 30.00 | G466V | 1397 G>T |
|  | | | KRAS | 19404 | 30.35 | A146T | 436G>A |
|  | | | TP53 | 10735 | 56.49 | R213Q | 638G>A |
|  | | | SMAD4 | 1151291 | 49.42 | G419V | 1256G>T |
| 2210396 | | | No hotspot |  |  |  |  |
| 2109351 | | | BRAF | 264460 | 30.40 | N581I | 1742A>T |
|  | | | TP53 | 10656 | 61.89 | R248W | 742C>T |
| 1608208 | | | PIK3CA | 763 | 23.9 | p.E545K | c.1633G>A |
|  | | | KRAS | 520 | 34.2 | p.G12V | c.35G>T |
| 390005 | | | APC | 18852 | 10.1 | p.R876* | c.2626C>T |
|  | | | KRAS | 521 | 8.6 | p.G12D | c.35G>A |
| 435776 | | | KRAS | 521 | 7.8 | p.G12D | c.35G>A |
|  | | | TP53 | 10726 | 10.5 | p.E286K | c.856G> |
|  | | | TP53 | 10911 | 27.4 | p.R283C | c.847C>T |
|  | | | ERBB2 | 14065 | 3.5 | p.V842I | c.2524G>A |
|  | | | TP53 | 43606 | 3.8 | p.G245D | c.734G>A |
|  | | | TP53 | 43692 | 3.2 | p.G154S | c.460G>A |
|  | | | TP53 | 43737 | 3.6 | p.C277Y | c.830G>A |
|  | | | TP53 | 43761 | 2.5 | p.E204= | c.612G>A |
|  | | | TP53 | 43962 | 3.1 | p.S269G | c.805A>G |
|  | | | TP53 | 43987 | 2.5 | p.D208N | c.622G>A |
|  | | | TP53 | 44194 | 5.8 | p.A84V | c.251C>T |
|  | | | TP53 | 44428 | 3.5 | p.N247= | c.741C>T |
|  | | | TP53 | 44705 | 3.3 | p.H233Y | c.697C>T |
|  | | | TP53 | 46284 | 5.3 | p.G279= | c.837G>A |
|  | | | TP53 | 99668 | 3.9 | p.R196* | c.586C>T |
|  | | | TP53 | 99933 | 3.1 | p.R273C | c.817C>T |
| 436027 | | | CTNNB1 | 5738 | 2.5 | p.A21T | c.61G>A |
|  | | | ERBB4 | 110095 | 3.1 | p.S341L | c.1022C>T |
|  | | | PTEN | 5149 | 3 | p.Q171* | c.511C>T |

Table S4. Relationship between common genetic alterations and clinicopathological factors of patients with SBA (N=48)

|  |  | ***APC*** | |  | ***CTNNB1*** | |  | ***KRAS*** | |  | ***TP53*** | |
| --- | --- | --- | --- | --- | --- | --- | --- | --- | --- | --- | --- | --- |
|  | No. | No. (%) | P |  | No. (%) | P |  | No. (%) | P |  | No. (%) | P |
| **Site** |  |  |  |  |  |  |  |  |  |  |  |  |
| Duodenum | 21 | 2 (10) | NS |  | 0 | NS |  | 4 (19) | NS |  | 6 (29) | 0.016 |
| Jejunum | 23 | 4 (17) |  |  | 3 (13) |  |  | 8 (35) |  |  | 16 (70) |  |
| Ileum | 4 | 1 (25) |  |  | 1 (25) |  |  | 3 (75) |  |  | 1 (25) |  |
| **Histologic type** |  |  |  |  |  |  |  |  |  |  |  |  |
| WD, MD | 37 | 7 (19) | NS |  | 2 (5) | NS |  | 14 (38) | NS |  | 18 (49) | NS |
| PD | 8 | 0 |  |  | 1 (13) |  |  | 1 (13) |  |  | 4 (50) |  |
| Mucinous | 3 | 0 |  |  | 1 (33) |  |  | 0 |  |  | 1 (33) |  |
| **Mucin phenotype** |  |  |  |  |  |  |  |  |  |  |  |  |
| Gastric type | 9 | 0 | NS |  | 0 | NS |  | 1 (11) | NS |  | 3 (33) | NS |
| Gastrointestinal type | 15 | 1 (7) |  |  | 1 (7) |  |  | 6 (40) |  |  | 4 (27) |  |
| Intestinal type | 23 | 6 (26) |  |  | 3 (13) |  |  | 8 (35) |  |  | 15 (65) |  |
| Null type | 1 | 0 |  |  | 0 |  |  | 0 |  |  | 1 (100) |  |
| **pT factor** |  |  |  |  |  |  |  |  |  |  |  |  |
| pT1-3 | 41 | 4 (10) | NS |  | 3 (7) | NS |  | 12 (29) | NS |  | 19 (46) | NS |
| pT4 | 7 | 3 (43) |  |  | 1 (14) |  |  | 3 (43) |  |  | 4 (57) |  |
| **Lymph node metastasis** |  |  |  |  |  |  |  |  |  |  |  |  |
| pN0 | 28 | 4 (14) | NS |  | 1 (4) | NS |  | 7 (25) | NS |  | 10 (36) | NS |
| pNx | 20 | 3 (15) |  |  | 3 (15) |  |  | 8 (40) |  |  | 13 (65) |  |
| **Distant metastasis** |  |  |  |  |  |  |  |  |  |  |  |  |
| Negative | 39 | 5 (13) | NS |  | 2 (5) | NS |  | 12 (31) | NS |  | 18 (46) | NS |
| Positive | 9 | 2 (22) |  |  | 2 (22) |  |  | 3 (33) |  |  | 5 (56) |  |
| **TNM stage** |  |  |  |  |  |  |  |  |  |  |  |  |
| l | 10 | 1 (10) | NS |  | 0 | NS |  | 4 (40) | NS |  | 2 (20) | NS |
| ll | 18 | 3 (17) |  |  | 1 (6) |  |  | 3 (17) |  |  | 8 (44) |  |
| lll | 11 | 1 (9) |  |  | 1 (9) |  |  | 5 (46) |  |  | 8 (73) |  |
| lV | 9 | 2 (22) |  |  | 2 (22) |  |  | 3 (33) |  |  | 5 (56) |  |
| **MMR status** |  |  |  |  |  |  |  |  |  |  |  |  |
| proficient | 40 | 4 (10) | NS |  | 4 (10) | NS |  | 13 (33) | NS |  | 18 (45) | NS |
| deficient | 8 | 3 (38) |  |  | 0 |  |  | 2 (25) |  |  | 5 (63) |  |

MD, moderately differentiated adenocarcinoma; MMR, DNA mismatch repair; mucinous, mucinous adenocarcinoma, PD; poorly differentiated adenocarcinoma; WD, well differentiated adenocarcinoma

NS; not significantly different
